# SARS-CoV-2 IgG seropositivity among people living with and without HIV in the first year of the COVID-19 pandemic in Haiti and Tanzania

**DOI:** 10.1016/j.ijregi.2026.100933

**Published:** 2026-06-18

**Authors:** Alexandra A. Cordeiro, Saidi Kapiga, Myung Hee Lee, Megan Willkens, Robert Peck, Benson Issarow, Nancy Dorvil, Kathleen F. Walsh, Stalz Charles Vilbrun, Vanessa Rouzier, Patrice Severe, Maureen M. Ward, Jean William Pape, Alexandra Appolon, Daniel Fitzgerald

**Affiliations:** 1 Department of Medicine, Center for Global Health, Weill Cornell Medicine, New York, NY, USA; 2 Mwanza Intervention Trials Unit, National Institute for Medical Research, Mwanza, Tanzania; 3 Department of Infectious Disease Epidemiology, London School of Hygiene & Tropical Medicine, London, UK; 4 Weill Bugando School of Medicine, Catholic University of Health and Allied Sciences, Mwanza, Tanzania; 5 The Haitian Group for the Study of Kaposi’s Sarcoma and Opportunistic Infections (GHESKIO), Port-au-Prince, Haiti

**Keywords:** COVID-19, SARS-CoV-2, Antibodies, Seroprevalence, HIV

## Abstract

**Objectives:**

People living with human immunodeficiency virus (PLWH) may have compromised humoral immune responses to severe acute respiratory syndrome coronavirus 2 (SARS-CoV-2), potentially influencing susceptibility to infection. Parallel comparative analyses of SARS-CoV-2 seropositivity and antibody levels were conducted among adults with and without human immunodeficiency virus (HIV) in Port-au-Prince, Haiti, and in Mwanza, Tanzania, early in the coronavirus disease 2019 (COVID-19) pandemic (2020-2021).

**Methods:**

In Haiti, participants were recruited from ongoing studies, including a community-based study of cardiovascular disease, a cohort of adults living with HIV, and a cohort with a history of tuberculosis. In Tanzania, participants were recruited from a cohort of adults with and without HIV. Demographic and clinical information was abstracted from electronic medical records. In Haiti, two blood samples were collected six months apart, at baseline and follow-up between September 2020 and September 2021. In Tanzania, a single blood sample was collected (August 2020-May 2021). Anti-SARS-CoV-2 antibodies were measured using the R&D COVID-SeroIndex Kantaro kit.

**Results:**

In Haiti, 383 PLWH and 484 individuals without HIV were enrolled. In Tanzania, 128 PLWH and 142 individuals without HIV were enrolled. In Haiti, overall SARS-CoV-2 positivity was lower among PLWH than among individuals HIV-uninfected (52.5% vs 65.0%; *P* < 0.001). Immunoglobulin G levels were also lower in PLWH (*P* < 0.001), with similar trends observed in the Tanzanian cohort (27.3% vs 51.4%).

**Conclusions:**

PLWH in Haiti and Tanzania had lower SARS-CoV-2 seropositivity and lower antibody levels than individuals without HIV, suggesting that some PLWH may mount weaker antibody responses to SARS-CoV-2.

## Highlights

• During the coronavirus disease pandemic, people living with human immunodeficiency virus (PLWH) in Haiti and Tanzania had lower serum immunoglobulin G antibodies.
• Severe acute respiratory syndrome coronavirus 2 antibodies rose faster in human immunodeficiency virus-uninfected compared to PLWH in Haiti.
• In Tanzania, seropositivity was lower in PLWH compared to human immunodeficiency virus-uninfected people.

## Introduction

Many population-based estimates have assessed the seroprevalence of anti-severe acute respiratory syndrome coronavirus 2 (anti-SARS-CoV-2) antibodies among people living with human immunodeficiency virus (PLWH) in high-income countries [[1], [2], [3], [4]]. However, seroprevalence data remain limited in low- and middle-income countries and have shown conflicting findings [5,6]. Seroprevalence studies among an adult population on antiretroviral therapy in India and Burkina-Faso found that PLWH had a higher prevalence of anti-SARS-CoV-2 immunoglobulin G (IgG) antibodies compared to individuals without human immunodeficiency virus (HIV) [7,8]. On the other hand, a cross-sectional study in South Africa found that PLWH who had not yet achieved virological suppression had lower SARS-CoV-2 antibody titers compared to controls without HIV [9]. Similarly, in a case-control study in the United States, PLWH were found to have lower IgG antibody levels compared to individuals without HIV [10]. Understanding SARS-CoV-2 seroprevalence and antibody responses among PLWH remains critical to improving public health responses to pandemic viruses in this population.

PLWH, particularly those with decreased CD4+ T-cell counts or unsuppressed HIV viremia, may have impaired humoral immune responses to SARS-CoV-2 infection due to chronic immune activation, B-cell dysfunction, and disrupted SARS-CoV-2-specific T-cell responses [[11], [12], [13]]. These immunologic alterations may influence both the magnitude and durability of antibody responses following SARS-CoV-2 infection. Several studies have compared the humoral and cellular immune responses elicited following SARS-CoV-2 infection among PLWH; however, seropositivity estimates and antibody levels have been inconsistent across studies [11,12].

These contrasting findings highlight the importance of characterizing SARS-CoV-2-specific immune response among PLWH. To address this gap, we aimed to compare the SARS-CoV-2 seroprevalence and antibody responses over 12 months among adults with and without HIV in Port-au-Prince, Haiti. Additionally, we examined whether similar patterns of SARS-CoV-2 seropositivity and antibody responses were observed among an independent cohort from Mwanza, Tanzania.

Haiti and Tanzania experienced synchronized SARS-CoV-2 epidemic waves, with initial case numbers in both countries peaking in mid-2020, followed by a secondary wave in early 2021 [14,15]. While public health mitigation measures and epidemic intensity differed between the two settings, national COVID-19 vaccination campaigns in both countries began in July 2021 [16,17]. In both settings, vaccination coverage remained low, approximately 3% in Haiti [18], and 2.8% in Tanzania by January 2022 [19]. The COVID-19 pandemic was further complicated by the generalized HIV epidemic in both countries, with an estimated HIV prevalence of 1.8% among adults (15-49 years) in Haiti and 4.5% in Tanzania [14]. The high prevalence of HIV in these countries raises important questions about how HIV status may influence SARS-CoV-2 seroprevalence among PLWH.

## Methods

### Study design and setting

This study was conducted at Groupe Haitien d’Etude du Sarcome de Kaposi des Infections Opportunistes (GHESKIO) center in Port-au-Prince, Haiti, and Bugando Medical Center in Mwanza, Tanzania. GHESKIO, founded in 1982, is a large medical center providing medical care for HIV, tuberculosis (TB), and other infectious diseases. Bugando Medical Center, situated along the shores of Lake Victoria in Mwanza city, is a tertiary hospital that oversees medical care for approximately 15 million people living in six regions in northwestern Tanzania.

### Study population Haiti

Participants were recruited from several ongoing longitudinal cohort studies at GHESKIO, including a population-based cohort of cardiovascular disease and clinical cohorts of HIV and TB. Comprehensive descriptions for each study have been published elsewhere [[20], [21], [22]]. Cohort members attending follow-up at GHESKIO were offered participation, and written informed consent was obtained. All participants were aged ≥18 years. Participants’ sociodemographic characteristics, health behaviors, clinical information, CD4 count, and HIV viral load data were abstracted from electronic medical records. Sociodemographic characteristics included age, sex, education, and income. A recent history of TB was defined as a documented, microbiologically confirmed TB diagnosis in the past 24 months. For the current study, two blood plasma samples were collected six months apart for anti-SARS-CoV-2 IgG antibody testing. Participants’ COVID-19 history was unknown at the time of enrollment. Participants were enrolled, and samples were collected between September 2, 2020, and September 9, 2021, at GHESKIO.

### Study population Tanzania

Participants were also recruited from an ongoing HIV and hypertension (HTN) cohort of 500 individuals with HIV and 500 without HIV, aged ≥18 years and <65 years, at Bugando Medical Center. Of the 1000 total participants from the cohort, 270 adults were randomly selected for anti-SARS-CoV-2 antibody testing. The study population and design of this ongoing cohort have been described elsewhere [23]. At study enrollment, blood pressure measurements, laboratory testing, and cardiovascular measurements were recorded, and information was collected on participant health behaviors, sociodemographic characteristics, and income status. Participants provided written informed consent before they participated in this COVID-19 study. A single sample was collected between August 24, 2020, and May 30, 2021, at Bugando Medical Center.

### Laboratory analysis

Circulating IgG antibodies against the SARS-CoV-2 spike (S) protein were measured in serum samples at Weill Cornell Medicine (New York) using a commercially available kit (COVID-SeroIndex, Kantaro Quantitative SARS-CoV-2 IgG antibody RUO Kit [R&D Systems, Bio-Techne, Minneapolis, MN]) according to the manufacturer’s instructions. This kit employs two direct enzyme-linked immunosorbent assays which allow for the detection of both IgG antibodies against the receptor-binding domain (RBD) peptide (expressed as a cut-off index [CI]) followed by a quantitative determination of IgG against full-length SARS-CoV-2 spike protein. Samples positive for both anti-RBD protein and anti-full-length spike protein were considered seropositive for COVID-19 antibodies per manufacturer’s instructions. Samples with discordant results were defined as samples positive for RBD peptide antibody and negative for spike protein antibody; these were classified as seronegative in the analyses.

### Statistical analysis

Descriptive statistics were reported for all participants across all cohort studies in Haiti and Tanzania. Proportions were reported for categorical variables, and medians with interquartile ranges for continuous variables by HIV status. Viral load at enrollment was summarized for the Haiti cohort, including median and proportion of participants with viral suppression (<1000 copies/mL). Seroprevalence estimates were reported as proportions with 95% Confidence Intervals and were restricted to seroprevalence at baseline (participants’ first blood draw). The Wilcoxon rank-sum test was used to compare antibody levels between people with and without HIV. An age-adjusted logistic regression model was used to compare seropositivity between HIV-uninfected and PLWH. Multivariable logistic regression models were used to evaluate associations between HIV status and SARS-CoV-2 seropositivity. Covariates included age, sex, level of education, income, and obesity/body mass index, based on data availability within each cohort. The proportion of participants with detectable SARS-CoV-2 antibodies was calculated monthly, and the trends over time were modeled using linear models. The Wilcoxon rank-sum test was used to assess differences in viral load by SARS-CoV-2 status among the Haiti cohort. All statistical analyses were performed in R (Version 4.2.3).

### Ethical considerations

Ethical approval for all study activities was provided by the Weill Cornell Medicine Institutional Review Board (Protocol 1506016328), the Tanzanian National Institute for Medical Research (Protocol NIMR/HQ/R.8c/Vol.1/1399), and the GHESKIO Research Ethics Committee (#1704018103; #1803019037 and #1401014658). Written informed consent was obtained from all study participants prior to participation in the study.

## Results

### Participant characteristics in Haiti

A total of 383 adults living with HIV and 484 individuals without HIV from Haiti participated in the study. Nearly all (95.3%) PLWH had a secondary school education or less, and half of the participants (53%) reported living on less than $50 (United States dollar) per month. A recent history of active TB was reported among 37 (7.6%) individuals without HIV and 64 (16.7%) PLWH. A total of 295 PLWH (77.3%) and 290 participants without HIV (68.8%) had two blood draws collected, with the first blood draw collected at baseline and the second collected at follow-up (Table 1).

**Table 1:** Characteristics of study participants by HIV status from several cohort studies at GHESKIO Centers, Port-au-Prince, Haiti and from the HIV and HTN cohort, Mwanza, Tanzania.

Characteristics	Haiti cohort		Tanzania cohort	
	HIV negative (N = 484)	HIV positive (N = 383)	HIV negative (N = 142)	HIV positive (N = 128)
**Sex**				
Female	340 (70.2%)	196 (51.2%)	96 (67.6%)	97 (75.8%)
Male	144 (29.8%)	187 (48.8%)	46 (32.4%)	31 (24.2%)
				
**Age (years)**				
Mean (SD)	45.7 (17.1)	39.6 (11.3)	36.5 (11.6)	39.0 (9.7)
Median [Min, Max]	45.0 [15, 84]	39.0 [18, 73]	36.5 (18, 59)	39 (20, 59)
**Education**				
Primary or less	195 (40.3%)	206 (53.8%)	115 (80.9%)	111 (86.7%)
Secondary	232 (47.9%)	159 (41.5%)	23 (16.2%)	14 (10.9%)
Tertiary or higher	43 (8.9%)	18 (4.7%)	4 (2.8%)	3 (2.3%)
Missing	14 (2.9%)	0 (0%)	—	—
**Civil status**				
Married/Cohabiting	184 (43.7%)	191 (49.9%)	92 (64%)	53 (41%)
Single	240 (49.6%)	123 (32.1%)	23 (16.2%)	14 (10.9%)
Divorced/Sep./Widowed	53 (12.6%)	69 (18.0%)	27 (19.0%)	61 (47.6%)
**Income (USD/month)**				
None	237 (49.0%)	133 (34.7%)	—	—
Less than $50	164 (33.9%)	70 (18.3%)	91 (64.1%)	81 (63.3%)
$50-200	51 (10.5%)	95 (24.8%)	40 (28.2%)	39 (30.5%)
>$200	23 (4.8%)	82 (21.4%)	10 (7.0%)	8 (6.3%)
Missing	9 (1.9%)	3 (0.8%)	1 (0.70%)	0 (0%)

Abbreviations: GHESKIO, Groupe Haitien d’Etude du Sarcome de Kaposi des Infections Opportunistes; HIV, human immunodeficiency virus; Hypertension (HTN); Max, maximum; Min, minimum; SD, standard deviation; USD, United States dollar.

### Participant characteristics in Tanzania

A total of 128 adult PLWH and 142 adults without HIV participated in the study. A total of 86 (31.9%) were classified as overweight or obese. Nearly all (86.7%) PLWH had a primary school education or less, and more than half (63.28%) reported living on less than $50 (United States dollar) per month (Table 1).

### SARS-CoV-2 seroprevalence in Haiti

**Figure 1:**
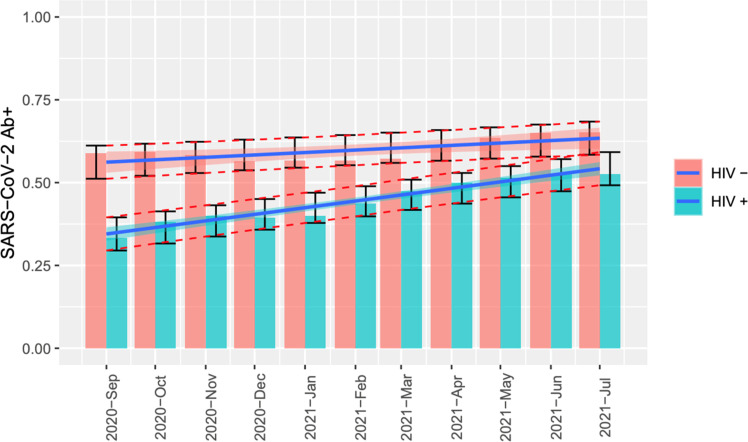
Cumulative incidence of SARS-CoV-2 antibodies among participants in Port-au-Prince, Haiti (September 2020 and July 2021). Incidence of SARS-CoV-2 antibodies was lower among PLWH compared to HIV-uninfected individuals. This figure displays the antibody levels of people who tested positive for SARS-CoV-2 antibodies. Data for those participants who tested negative for SARS-CoV-2 were not included in this analysis. Abbreviation: HIV, human immunodeficiency virus; PLWH people living with HIV; SARS-CoV-2, severe acute respiratory syndrome coronavirus 2

At the initial blood draw (baseline sample collection), SARS-CoV-2 seroprevalence was 57.0% (276/484) among individuals without HIV and 39.2% (150/383) among PLWH. Among individuals without HIV, the proportion of participants with detectable SARS-CoV-2 antibodies increased from 58.8% in September 2020 to 65.0% at the end of July 2021. During the same period, among PLWH, the proportion of SARS-CoV-2 antibodies increased from 33.3% to 52.5% (Figure 1).

### SARS-CoV-2 seroprevalence in Tanzania

Of the 270 participants enrolled in the study, 108 (40.0%) were SARS-CoV-2 seropositive. Among PLWH, 35 (27.3%) were seropositive compared to 73 (51.4%) of individuals without HIV (*P* < 0.001). As in Haiti, the levels of IgG anti-SARS-CoV-2 antibodies for the spike protein were significantly lower in PLWH compared with individuals without HIV in Tanzania (*P* = 0.049).

### SARS-CoV-2 antibody levels in Haiti

**Figure 2:**
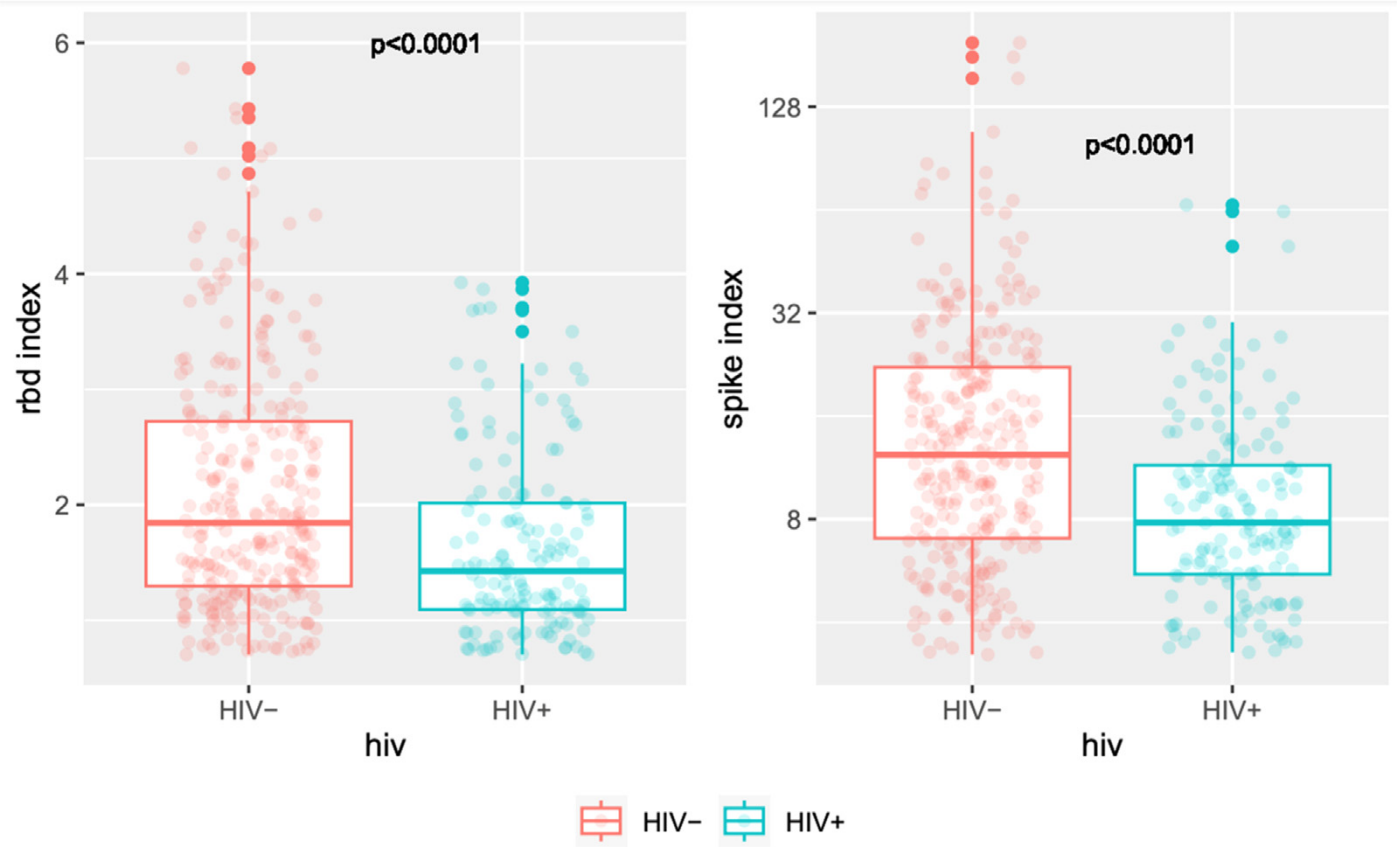
Comparison of SARS-CoV-2 receptor-binding domain (RBD) and spike protein antibody levels among HIV-uninfected individuals (red) and PLWH (blue), in Port-au-Prince, Haiti (September 2020 and July 2021). Antibody levels are notably higher among HIV-uninfected individuals. Analyses were restricted to the subset of individuals who tested positive for SARS-CoV-2 on both ELISA assays at the first blood draw. Abbreviations: ELISA, enzyme-linked immunosorbent assay; HIV, human immunodeficiency virus; PLWH, people living with HIV; RBD, receptor-binding domain; SARS-CoV-2, severe acute respiratory syndrome coronavirus 2.

The levels of IgG anti-SARS-CoV-2 antibodies were significantly lower in PLWH compared with HIV-uninfected individuals (*P* <0.001) (Figure 2). Among 194 PLWH who tested positive after the initial serology test against the RBD peptide, 44 (22.7%) had discordant SARS-CoV-2 results with a negative test for anti-spike protein antibody. Among HIV-uninfected individuals who tested positive on the initial serology test (n = 324), 48 (14.8%) had discordant SARS-CoV-2 serology results, defined as a sample positive for RBD peptide antibody but negative for spike protein antibody. Chi-squared test demonstrated a significant difference in discordant test results (*P* = 0.023).

### SARS-CoV-2 antibody levels in Tanzania

Antibody levels for RBD did not differ significantly by HIV status in Tanzania (*P* = 0.489). Out of the entire cohort, 12 (4.4%) PLWH and 7 (2.6%) participants without HIV had discordant SARS-CoV-2 serology results (*P* = 0.048). For these participants, RBD tests for SARS-CoV-2 antibodies were positive, but confirmatory anti-spike antibody tests were negative.

### Associated risk factors of SARS-CoV-2 seropositivity in Haiti

In addition to HIV status, older age was also significantly associated with higher rates of seropositivity (odds ratio = 1.10, 95% confidence interval 1.01-1.21, *P* = 0.039) for a 10-year increase in age. No statistically significant association was noted between SARS-CoV-2 seropositivity and other baseline factors. In multivariate analysis, controlling for age did not change the association between HIV status and seropositivity.

### Associated risk factors of SARS-CoV-2 seropositivity in Tanzania

In the Tanzanian cohort, multivariable analysis demonstrated that PLWH had lower odds of SARS-CoV-2 seropositivity after adjusting for obesity. Participants classified as overweight or obese had higher SARS-CoV-2 seroprevalence compared to those with normal weight (53.5% vs 33.7%, *P* = 0.002). There was no association observed between SARS-CoV-2 seropositivity and age, sex, level of education, or income.

### Association of HIV clinical markers and SARS-CoV-2 antibody responses in Haiti

**Figure 3:**
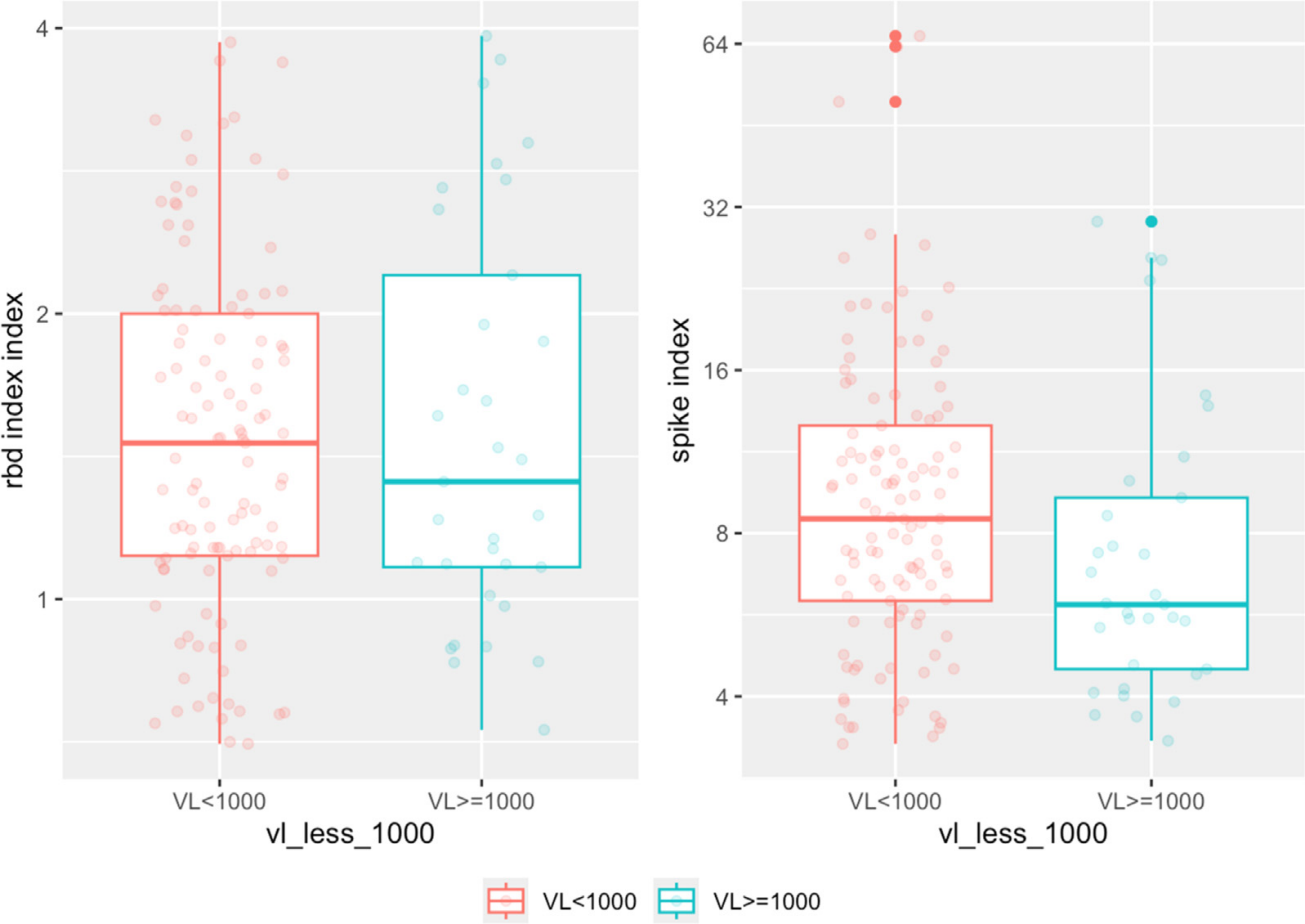
SARS-CoV-2 RBD and spike antibody levels by viral suppression status among seropositive PLWH in Port-au-Prince, Haiti (September 2020 and July 2021). Abbreviations: PLWH, people living with HIV; RBD, receptor-binding domain; SARS-CoV-2, severe acute respiratory syndrome coronavirus 2.

Among the 383 PLWH in Haiti, we analyzed clinical markers of HIV disease (i.e., CD4+ count, viral load, viral suppression) that were associated with SARS-CoV-2 antibody responses. Viral load data were available for 378 participants. Median viral load at enrollment was 39.5 copies/mL, and 70% were virally suppressed (viral load < 1000). Viral load tended to be lower among individuals who were SARS-CoV-2 seropositive compared to individuals who were seronegative; however, this difference did not reach significance (*P* = 0.13). Among SARS-CoV-2 seropositive PLWH, antibody responses were further examined by HIV viral suppression status. Antibody levels for RBD did not differ by viral suppression status; however, spike antibody levels were significantly lower for participants with unsuppressed viral load (viral load ≥1000 copies/mL) compared to virally suppressed individuals (5.9 vs 8.5, *P* = 0.03) (Figure 3). These findings suggest that higher HIV viral loads may be associated with a diminished humoral immune response to SARS-CoV-2.

## Discussion

Our data demonstrate that PLWH in Haiti and Tanzania had lower SARS-CoV-2 seropositivity and lower antibody levels compared to individuals without HIV. This study used samples that were collected during the first year of the COVID-19 pandemic, before the availability of the COVID-19 vaccine. Our study results suggest that some PLWH living in lower-income countries, such as Haiti and Tanzania, may develop lower detectable anti-SARS-CoV-2 antibody responses compared to individuals without HIV. Chronic immune activation, CD4+ T-cell dysregulation, and impaired B-cell responses may reduce the magnitude or durability of antibody responses following SARS-CoV-2 infection, particularly among individuals with unsuppressed HIV viremia.

In reviewing the literature, we identified nine studies that enrolled PLWH and adults without HIV who tested positive by polymerase chain reaction for SARS-CoV-2 infection and were then followed during convalescence for 2-6 months, with serum or plasma tested for IgG antibodies to the spike or RBD antigen of SARS-CoV-2. The assays to detect antibodies differed from study to study, and most were in-house developed serologic assays. These were relatively small studies, with an average of 20-30 PLWH and a similar number of people without HIV enrolled. In five of these studies, no difference in IgG antibody test positivity or antibody levels against SARS-CoV-2 antigens were detected [24], [25], [26], [27]. Of note, in four of these five studies, all people with HIV were on antiretroviral therapy and were HIV virologically suppressed [[11], [18], [25], [26], [27]]. In the fifth study, at least 85% were virologically suppressed [24]. In contrast, four other studies demonstrated an association between HIV status and low antibody levels against SARS-CoV-2 antigens [[28], [29], [30]]. In these studies, PLWH had significantly lower rates of antibody test positivity and lower levels of antibodies. Of note, these four cohorts had high rates of HIV viremia, with one study reporting detectable viremia in 60% of participants with HIV [29]. Two of these positive studies also showed that people with HIV viremia had lower SARS-CoV-2 test positivity and lower levels of antibodies against SARS-CoV-2 than people with HIV who were on antiretroviral therapy (ART) and virologically suppressed [28,29]. One study also found the lowest levels of antibodies against SARS-CoV-2 antigens were among PLWH with asymptomatic or mild COVID-19 disease [[28], [29], [30], [31]]. In summary, HIV cohorts with a higher proportion of PLWH experiencing HIV viremia and a greater number of asymptomatic or mild COVID-19 disease, in both high- and low-income settings, are likely to have lower seropositivity and lower IgG levels against SARS-CoV-2.

In our literature review, we also found five serologic studies in asymptomatic healthy PLWH designed to determine the prevalence of antibodies against SARS-CoV-2 [1,9,10,[28], [29], [30], [31], [32], [33]]. Four of the studies also had HIV-negative controls [9,10,34]. Three studies demonstrated that PLWH have a lower prevalence of a positive antibody test against SARS-CoV-2 Spike or RBD antigen and/or that low test positivity and antibody titers were associated with PLWH with detectable HIV viremia [32]. In the two negative studies that did not show an association between HIV status and anti-SARS-CoV-2 antibodies, one had a very small sample size [33] and the second tested for anti-SARS-CoV-2 nuclear antigen antibody [1], which has a low sensitivity and a rapid decline in titer post-infection compared to anti-spike or RBD antibodies. PLWH represent a heterogeneous population, and SARS-CoV-2 immune responses may vary according to CD4+ cell count, viral suppression, ART adherence, and duration of HIV infection. Consistent with this heterogeneity, in the Haitian cohort, we observed a significant difference in spike antibody levels among PLWH with unsuppressed HIV viral load compared to virally suppressed individuals, suggesting that HIV may influence responses to SARS-CoV-2 infection.

In summary, according to our literature review, both the studies of people with a history of prior polymerase chain reaction-positive COVID-19 and studies of healthy community members with and without HIV suggest that IgG anti-SARS-CoV-2 antibody test positivity and IgG titers are lower in PLWH than in adults without HIV, especially for PLWH with detectable HIV viremia. Consistent with this literature, among SARS-CoV-2 seropositive PLWH in our Haiti cohort, spike antibody levels were lower among individuals with unsuppressed HIV viral load, while RBD antibody levels were not associated with viral load. We did not have plasma HIV-1 ribonucleic acid levels for the participants in this study at the time we tested for SARS-CoV2 antibodies. However, other studies suggest that 26% of PLWH on ART in Haiti [28] and 12% in Tanzania [35] have detectable HIV-1 ribonucleic acid in plasma. These rates of virologic failure may be sufficient to decrease the prevalence and incidence of SARS-CoV-2 antibodies in these settings.

### Strengths and limitations

A primary strength of this study was the evaluation of SARS-CoV-2 seroprevalence and antibody response during the pre-vaccination period among PLWH and individuals without HIV in both Haiti and Tanzania. By enrolling participants during this timeframe, our results reflect natural infection rather than vaccine-induced immunity, removing a potential confounder. Several limitations should also be considered. First, the observational design limits our ability to determine causal relationships. Second, our study was conducted in two large urban clinics in Haiti and Tanzania, which may limit direct comparability across cohorts as well as generalizability of these findings to the broader population of PLWH in both countries. Various factors, including demographics and access to COVID-19 testing facilities, may also impact the overall prevalence of SARS-CoV-2 in other regions. Future longitudinal studies are needed to explore the relationship between immune status, including vaccine-induced memory and prior infection, on SARS-CoV-2 seropositivity among PLWH.

## Conclusions

Our study confirms that PLWH in Haiti and Tanzania had lower SARS-CoV-2 seropositivity and lower SARS-CoV-2 antibody levels compared to individuals without HIV in the first year of the COVID-19 pandemic. These findings are consistent with other studies of PLWH, particularly for populations of PLWH with higher rates of HIV viremia. These results suggest that PLWH in routine care in low-resource settings may not mount as high an antibody response against SARS-CoV-2 compared to people without HIV. Therefore, PLWH may benefit from targeted prevention programs - such as improved symptom monitoring, aggressive vaccination campaigns, and testing programs– in the current and future pandemics.

## CRediT authorship contribution statement

**Alexandra A. Cordeiro:** Data curation, Formal analysis, Writing – original draft, Writing – review & editing. **Saidi Kapiga:** Methodology, Supervision, Writing – review & editing. **Myung Hee Lee:** Data curation, Formal analysis, Writing – review & editing, Visualization. **Megan Willkens:** Data curation, Writing – review & editing. **Robert Peck:** Funding acquisition, Methodology, Project administration, Supervision, Writing – original draft, Writing – review & editing. **Benson Issarow:** Data curation, Writing – review & editing. **Nancy Dorvil:** Data curation, Writing – review & editing. **Kathleen F. Walsh:** Writing – review & editing. **Stalz Charles Vilbrun:** Writing – review & editing. **Vanessa Rouzier:** Project administration, Supervision, Writing – review & editing. **Patrice Severe:** Project administration, Writing – review & editing. **Maureen M. Ward:** Investigation, Writing – review & editing. **Jean William Pape:** Conceptualization, Supervision, Writing – review & editing. **Alexandra Appolon:** Writing – review & editing. **Daniel Fitzgerald:** Conceptualization, Funding acquisition, Methodology, Supervision, Writing – original draft, Writing – review & editing.

## Declaration of competing interest

The authors have no competing interests to declare.
